# Effect of CO_2_ Partial Pressure on the Corrosion Inhibition of N80 Carbon Steel by Gum Arabic in a CO_2_-Water Saline Environment for Shale Oil and Gas Industry

**DOI:** 10.3390/ma13194245

**Published:** 2020-09-23

**Authors:** Gaetano Palumbo, Kamila Kollbek, Roma Wirecka, Andrzej Bernasik, Marcin Górny

**Affiliations:** 1 Department of Chemistry and Corrosion of Metals, Faculty of Foundry Engineering, AGH University of Science and Technology, 30-059 Krakow, Poland; 2 Academic Centre for Materials and Nanotechnology, AGH University of Science and Technology, Mickiewicza St. 30, 30-059 Kraków, Poland; kamila.kollbek@agh.edu.pl (K.K.); roma.wirecka@fis.agh.edu.pl (R.W.); 3 Department of Condensed Matter Physics, Faculty of Physics and Applied Computer Science, AGH University of Science and Technology, Mickiewicza St. 30, 30-059 Krakow, Poland; bernasik@agh.edu.pl; 4 Department of Cast Alloys and Composites Engineering, Faculty of Foundry Engineering, AGH University of Science and Technology, 30-059 Krakow, Poland; mgorny@agh.edu.pl

**Keywords:** high-pressure CO_2_ corrosion, corrosion inhibition, gum arabic, carbon steel N80

## Abstract

The effect of CO_2_ partial pressure on the corrosion inhibition efficiency of gum arabic (GA) on the N80 carbon steel pipeline in a CO_2_-water saline environment was studied by using gravimetric and electrochemical measurements at different CO_2_ partial pressures (e.g., $P_{{\mathrm{CO}}_{2}}$ = 1, 20 and 40 bar) and temperatures (e.g., 25 and 60 °C). The results showed that the inhibitor efficiency increased with an increase in inhibitor concentration and CO_2_ partial pressure. The corrosion inhibition efficiency was found to be 84.53% and 75.41% after 24 and 168 h of immersion at $P_{{\mathrm{CO}}_{2}}$ = 40 bar, respectively. The surface was further evaluated by scanning electron microscopy (SEM), energy dispersive spectroscopy (EDS), grazing incidence X-ray diffraction (GIXRD), and X-ray photoelectron spectroscopy (XPS) measurements. The SEM-EDS and GIXRD measurements reveal that the surface of the metal was found to be strongly affected by the presence of the inhibitor and CO_2_ partial pressure. In the presence of GA, the protective layer on the metal surface becomes more compact with increasing the CO_2_ partial pressure. The XPS measurements provided direct evidence of the adsorption of GA molecules on the carbon steel surface and corroborated the gravimetric results.

## 1. Introduction

Shale oil and gas are “unconventional” resources of natural oil and gas trapped in fine-grained sedimentary rocks called shale. The rapid expansion of shale oil and gas exploration and the development of a new technology (i.e., hydraulic fracturing (HF) techniques), has seen the popularity of these natural resources to grow over the years. However, after years of exploitation, the oil and gas production in the reservoir declines to result in a major economic challenge for the oil companies. The injection of CO_2_ at high pressure into the wellbore is an effective method to increase the oil fields lifetime [1,2,3,4]. This process is usually referred to as carbon dioxide flooding enhanced oil recovery (CO_2_-EOR). However, CO_2_ gas dissolves in the fluid to form the weak carbonic acid, which in turn dissociates into bicarbonate and carbonate anions [4,5]. The presence of this weak acid can lead to severe corrosion attacks on the steel structures [4,5,6].

Another common problem encountered in the extraction of these natural resources is the use of aggressive fluids with high concentrations of chloride ions (e.g., fracturing fluid) [7]. In the HF process, the fluid usually injected into the wellbore is a neutral water-based chloride solution (up to 4% of potassium chloride) with different additives (i.e., inhibitors of scaling, thickening agents, corrosion inhibitors, etc.) [8]. The literature reports that the presence of a high concentration of chloride ions in a CO_2_-containing fluid can exponentially accelerate the dissolution of the steel [6,9].

Carbon and low-alloys steel are often used in the construction of the pipeline in the shale oil and gas industry infrastructures, mainly due to its durability, ductility, high strength, and low cost [7,8,10]. However, due to these harsh operating conditions encountered during the exploitation of these natural resources, the steel is prone to corrode. One practical and relatively cheap method for controlling sweet corrosion in the shale oil and gas industry is the use of corrosion inhibitors. Corrosion inhibitors are substances that added to the solution greatly reduce the dissolution of the metal by forming a protective layer on its surface. The literature reports that over the last decades the use of corrosion inhibitors as a means to mitigate CO_2_ corrosion that occurs inside the carbon steel pipelines has received a wide interest. Nitrogen-based compounds such as pyridine derivatives [11] imidazolines [12], benzimidazole derivatives [13], and amines [14] were found to be effective corrosion inhibitors against CO_2_ corrosion. However, most of these compounds are reported to be toxic and their synthesis can be very expensive [15,16]. These drawbacks and the increase in environmental awareness have led many researchers to focus on the use of more naturally occurring substances as corrosion inhibitors. Plant extracts substances, such as berberine extract [17], *Momordica charantia* [18], *Gingko biloba* [19] were successfully tested as green corrosion inhibitors in CO_2_-saturated saline solutions.

The last trend of research has also seen the use of many naturally occurring polymers as green corrosion inhibitors in various corrosive environments [10,20,21,22]. They are abundant in nature, environmentally sustainable, and have an appreciable solubility. Additionally, polymers, unlike small molecules, with their multiple adsorption sites for bonding on the metal surface, are expected to show a higher corrosion inhibition efficiency, compared to their monomer counterpart.

Umoren et al. [15] studied the corrosion inhibition effect of two naturally occurring polymers such as carboxymethyl cellulose and chitosan for API 5 L X60 steel in a CO_2_ saline solution at $P_{{\mathrm{CO}}_{2}}$ = 1 bar. The results showed that both inhibitors reduced the corrosion rate of the metal due to the formation of a protective layer on its surface. Singh et al. [23] studied the corrosion inhibition effect of a modified natural polysaccharide (e.g., guar gum + methylmethacrylate) in a 3.5 wt% NaCl solution saturated with CO_2_ (e.g., $P_{{\mathrm{CO}}_{2}}$ = 1 bar) at 50 °C. The authors found that this modified polysaccharide acted like a good corrosion inhibitor for P110 steel with maximum inhibition efficiency found to be 90%. However, most of these studies were carried out at atmospheric pressure (e.g., $P_{{\mathrm{CO}}_{2}}$ = 1 bar). The CO_2_-EOR process can significantly increase the dissolution of the tube. As reported by many studies, the severity of the CO_2_ corrosion attack increases with an increase in CO_2_ partial pressure due to the increase in acidity of the fluid [4,5,6]. Therefore, understanding how the CO_2_ partial pressure can influence the inhibitory action of certain corrosion inhibitors in CO_2_ saline environments is important and can help to minimize the material and economic losses.

Mustafa et al. [4] studied the effect of the CO_2_ partial pressure (e.g., 10, 40, and 60 bar) on the corrosion inhibition of an imidazoline-based inhibitor for X52 steel exposed to CO_2_ water saline solution at 60 °C. The authors reported that the inhibitor efficiency of the tested inhibitor was observed to be strongly affected by the concentration of inhibitor and CO_2_ partial pressure. Ansari et al. [16] studied the influence of a modified chitosan corrosion inhibitor on J55 carbon steel in a 3.5 wt% NaCl solution saturated with CO_2_ at 60 bar and 65 °C, reporting a corrosion inhibition efficiency of 95%. Yet, all inhibitors tested so far are labeled either as toxic or are expensive to synthesize.

Gum arabic (GA) is a natural polymer obtained from the Acacia trees of the Leguminosae family [22] and it has been reported to successfully inhibit the corrosion of the steel in different environments [7,21,22,24,25,26,27,28]. Furthermore, GA is often used in the fracturing fluid as a thickening agent to increase the viscosity of the fluid [29]. Therefore, due to the encouraging results presented by these studies and the continuous research of affordable and eco-friendly corrosion inhibitors, this work was undertaken to study the efficacy of GA as an eco-friendly corrosion inhibitor to mitigate high-pressure CO_2_ corrosion for carbon steel pipeline in a saline solution. This paper also aims to show that GA not only can be used as a thickening agent in the make-up of the fracturing fluid, but it could also be used as an active component in corrosion inhibitor in the shale gas industry. To this end, the study was performed in an autoclave in the presence and absence of different concentrations of GA, different CO_2_ partial pressures, and different temperatures using weight loss and electrochemical measurements. SEM-EDS, GIXRD, and XPS measurements were also employed to characterize the corrosion product layer and to support the gravimetric and electrochemical results.

## 2. Experimental Procedure

### 2.1. Materials

**Figure 1:**
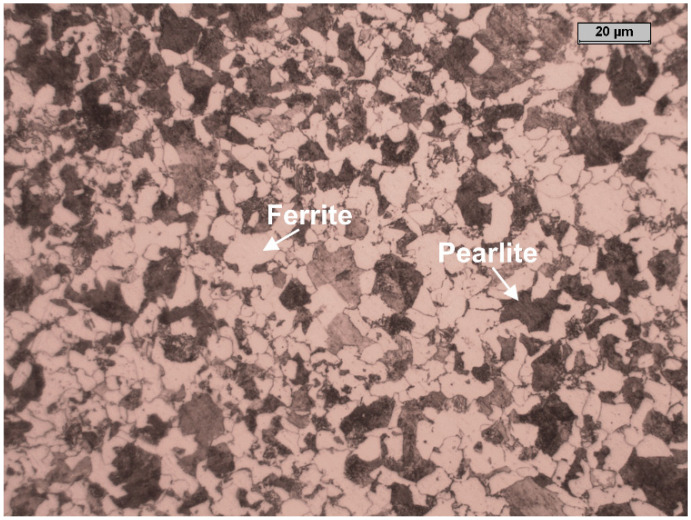
Optical micrographs of the N80 carbon steel microstructures.

The study was carried out on carbon steel (N80) with composition of (weight %): C 0.39%, Mn 1.80%, Si 0.26%, Cu 0.26%, V 0.19%, Cr 0.04%, Ni 0.04%, Al 0.03%, Mo 0.003%, Co 0.002%, Sn 0.004%, S 0.001%, P 0.001% and the remainder Fe. Figure 1 shows that the microstructure of the N80 carbon steel pipeline is composed of perlite and ferrite (α-Fe) phases, where the latter phase accounting for circa 41% of the total. The samples used in this study were machined from pipeline carbon steel, ground with silicon carbide abrasive paper up to 1200 grit, then were ultrasonically washed with distilled water, dried with absolute alcohol.

All experiments were carried out in 3% of potassium chloride (KCl). Potassium salt was used in this study instead of the more common NaCl, because in the fracturing fluid, the potassium (K^+^) ions formed a semi-permeable membrane on the shale rock and therefore, preventing the water from entering the shale.

The tested solution was prepared from reagent grade material potassium chloride (Sigma-Aldrich) and pure deionized water with an electrical resistivity of 0.055 µS/cm at T = 25 °C. The tested inhibitor was purchased from Sigma-Aldrich (Warsaw, Poland). The concentrations of inhibitor solution prepared and used for the study ranged from 0.6 up to 2.0 g L^−1^.

### 2.2. Gravimetric Measurements

The gravimetric experiments were carried out in a 1.2 L high-pressure autoclave (PARR instrument) at different CO_2_ partial pressures (1, 20, and 40 bar). The coupons were suspended in a 1.0 L solution in the presence and absence of different concentrations of the inhibitor (i.e., from 0.6 up to 2.0 g L^−1^) at 25 and 60 °C. Before each experiment, the tested solution was deaerated with CO_2_ for 2 h under atmospheric pressure and then CO_2_ was purged for another 2 h at the tested pressure after the introduction of the samples. After saturation, the pH and conductivity of the tested solution were 4.5 and 60.30 mS cm^−1^ at 1 bar and 25 °C, respectively. To ensure homogeneous mixing, a Teflon-coated blade agitator was used (e.g., 200 rpm). The weight loss was determined by retrieving the coupons after 24 h of immersion by means of an analytical balance with an accuracy of ±0.1 mg. To assess the effect of time, the samples were immersed for 168 h in the presence and absence of 1.0 g L^−1^ of GA at different CO_2_ partial pressures (1, 20, and 40 bar). The corrosion products were removed according to the ASTM G1-90 [30], then the specimens were ultrasonically washed with distilled water, dried with absolute alcohol, and reweighed. In each case, the experiment was conducted thrice and the corrosion rate (*CR*) in mm y^−1^ was obtained from the following equation:

(1) $$CR\;({\mathrm{mm}\;y}^{-1})=\frac{87.6\;\Delta m}{dAt}$$

where, Δ*m* is the weight loss calculated form the difference between the initial (*W*_i_) and the final (*W*_f_) weight (mg). d is the density (7.87 g cm^−3^), *A* is the surface of the sample (cm^−2^) and *t* is the immersion time (h). The inhibition efficiency (*IE*%) was determined using the following equation [20,22,26]:

(2) $$IE\%=\frac{CR-{CR}^{\mathrm{inh}}}{CR}\times 100$$

where *CR*^inh^ and *CR* are the corrosion rates of the steel with and without the inhibitor, respectively.

### 2.3. Electrochemical Experiments

The electrochemical experiments were carried out in a 1.2 L high-pressure autoclave (PARR instrument) at different CO_2_ partial pressures (1, 20, and 40 bar) with a conventional three-electrode system. N80 carbon steel specimen was used as a working electrode, a platinum foil as a counter electrode (CE), and a high-pressure 0.1 M KCl Ag/AgCl probe was used as a reference electrode. To ensure homogeneous mixing, a Teflon-coated blade agitator was used (200 rpm). The electrochemical impedance spectroscopy (EIS) and potentiodynamic polarization (PDP) measurements were carried in a Gamry reference 600 potentiostat/galvanostat electrochemical system after the sample was exposed for 24 h in the tested solution, with and without the presence of 1.0 g L^−1^ of GA. The EIS tests were performed over the frequency range of 100 kHz to 10 mHz and amplitude of 10 mV at open circuit potential. The data were then fitted by means of Echem Analyst 5.21 software using the opportune equivalent circuit. The *IE*% was calculated from the polarization resistances (*R*_p_) determined from the fitting process using the following equation [21,26]:

(3) $$IE\%=\frac{R_{p}^{\mathrm{inh}}-R_{p}}{R_{p}^{\mathrm{inh}}}\times 100$$

where *R*_p_^inh^ and *R_p_* are the values of the polarization resistances in the presence and absence of the inhibitor, respectively. The PDP measurements were carried out at a potential of ±−0.3 V from the OCP and a scan rate of 1 mV s^−1^. The potentiodynamic parameters were determined by means of Echem Analyst 5.21 software. The values of *IE*% were calculated from the measured *i*_corr_ values using the relationship [21,26]:

(4) $$IE\%=\frac{i_{\mathrm{corr}}-i_{corr}^{inh}}{i_{\mathrm{corr}}}\times 100$$

where $i_{\mathrm{corr}}$ and $i_{\mathrm{corr}}^{\mathrm{inh}}$ represent the values of the corrosion current densities without and with inhibitor, respectively.

### 2.4. Surface Analysis

The surface of the samples, prepared as described above, were analyzed in the presence and absence of 1.0 g L^−1^ of GA. After the immersion, the samples were removed and rinsed with deionized water and dried. The surface analysis was carried out by means of different techniques such as a scanning electron microscopy combined with an energy dispersive spectroscopy, grazing incidence X-ray diffraction (GIXRD), and an X-ray photoelectron spectroscopy (XPS). The SEM measurements were carried out by using a JEOL scanning electron microscope. The GIXRD analysis was carried out to further determine the composition of the corrosion products film. Grazing incident X-ray diffraction (GIXRD) with an incident angle of 3° was applied to study samples phase composition. A Panalytical Empyrean X-ray diffractometer in the parallel beam geometry (Goebel mirror in the incident beam optics and parallel plate collimator in the secondary beam optics) with Co lamp (Kα = 1.7902 Å) was used to perform measurements. The samples were scanned with a 0.02° step in the range of 20°– 70° at room temperature. The XPS analysis was carried out in a PHI 5000 VersaProbe II spectrometer with an Al Kα monochromatic X-ray beam. The X-ray source was operated at 25 W and 15 kV beam voltages. Dual-beam charge compensation with 7 eV Ar^+^ ions and 1 eV electrons was used to maintain a constant sample surface potential regardless of the sample conductivity. The pass energy of the hemispherical analyzer for the iron (Fe 2p) spectra was fixed at 23.5 eV and for other elements at 46.95 eV. The spectra were charge corrected to the mainline of the carbon C 1 s spectrum set to 284.8 eV.

## 3. Results and Discussion

### 3.1. Effect of Pressure and Temperature

#### 3.1.1. Gravimetric Experiments

**Figure 2:**
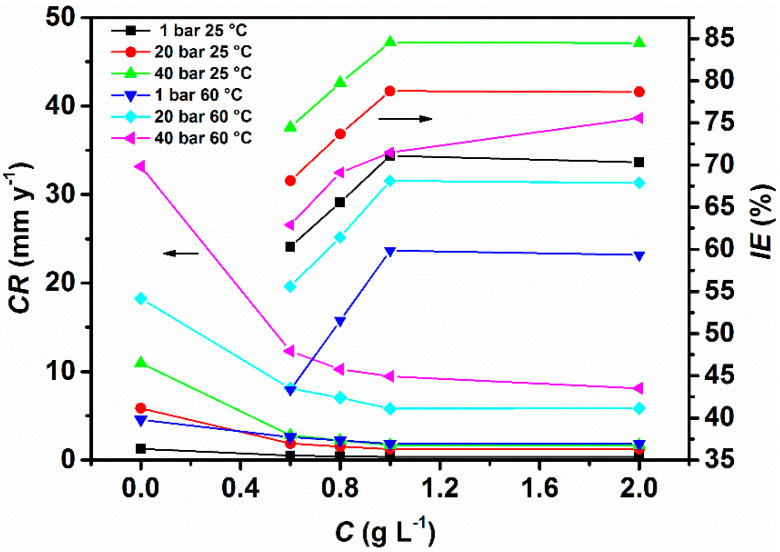
Corrosion inhibitor efficiency at different concentrations of gum arabic (GA) and CO_2_ partial pressures after 24 h of immersion.

Figure 2 and Table S1 show the corrosion rate and the variation of the inhibition efficiency obtained at different concentrations of GA and CO_2_ partial pressures. It follows from the data that *CR* increases with an increase of CO_2_ partial pressure, going from 1.28 to 10.95 mm y^−1^ at $P_{{\mathrm{CO}}_{2}}$ = 1 bar and $P_{{\mathrm{CO}}_{2}}$ = 40 bar at 25 °C, respectively.

The solubility of CO_2_ in water increases sharply with increasing the pressure of the system [31]. The high corrosion rate observed at higher CO_2_ partial pressures can be explained with the increase of the acidity of the solution. In fact, in the presence of CO_2_, the weak carbonic acid is formed, which in turn dissociates in ${\mathrm{HCO}}_{3}^{-}$ and in ${\mathrm{CO}}_{3}^{2-}$, according to the following reactions:

(5) $${\mathrm{CO}}_{2}+H_{2}O↔H_{2}{\mathrm{CO}}_{3}$$

(6) $$H_{2}{\mathrm{CO}}_{3}↔H^{+}+{\mathrm{HCO}}_{3}^{-}$$

(7) $${\mathrm{HCO}}_{3}^{-}↔H^{+}+{\mathrm{CO}}_{3}^{2-}$$

The corrosion process in a CO_2_ containing solution is controlled by the anodic reaction (Equation (8)) and the three cathodic reactions (Equation (9)–(11)) [4,5]:

(8) $$\mathrm{Fe}\to {\mathrm{Fe}}^{2+}+{2e}^{-}$$

(9) $${2H}_{2}{\mathrm{CO}}_{3}+{2e}^{-}\to H_{2}+{2\mathrm{HCO}}_{3}^{-}$$

(10) $${2\mathrm{HCO}}_{3}^{-}+{2e}^{-}\to H_{2}+{2\mathrm{CO}}_{3}^{2-}$$

(11) $${2H}^{+}+{2e}^{-}\to H_{2}$$

The pH of the solution plays an important role in determining the corrosion rate of carbon steel in a CO_2_ environment. As the CO_2_ partial pressure increases, its solubility also increases, resulting in an increase of the carbonic acid concentration in the solution (Equation (5)). Nesic’ predicted that the concentrations of H_2_CO_3_ in the solution would increase of about 40 times with changing the pressure from $P_{{\mathrm{CO}}_{2}}$ = 1 bar to $P_{{\mathrm{CO}}_{2}}$ = 40 bar [31]. Increasing the concentration of carbonic acid leads to an increase in the rate of reduction of carbonic acid and bicarbonate ions (Equations (9) and (10)), and ultimately the anodic dissolution of the steel (Equation (8)) as reported by several studies [4,5,31].

After the addition of the inhibitor, it can be seen that the corrosion rate of the metal is greatly reduced going from 1.28 to 0.37 mm y^−1^, with a maximum corrosion inhibition efficiency found to be 71.09% at $P_{{\mathrm{CO}}_{2}}$ = 1 bar, after 24 h of immersion. The data shows that in contrast to the uninhibited solution, an increase in CO_2_ partial pressure has a favorable effect on the corrosion rate of the metal in the presence of the inhibitor. It follows from Figure 2 that *IE*, which varies inversely with *CR,* significantly increased after the addition of GA and with CO_2_ partial pressure, with a maximum corrosion inhibition efficiency of 78.77% and 84.53% at $P_{{\mathrm{CO}}_{2}}$ = 20 bar and $P_{{\mathrm{CO}}_{2}}$ = 40 bar, after 24 h of immersion, respectively [4].

The literature reports that GA [7,21], and in general polysaccharides-like inhibitors [15,20], is mainly adsorbed on the metal surface in acidic condition by weak electrostatic interaction between the protonated inhibitor molecules and the chloride ions adsorbed on the metal surface. In a weak acid solution GA molecules are in equilibrium with their protonated molecules according to the following reaction (see also Section 3.5.1) [7]:

(12) $$\mathrm{GA}+{\mathrm{xH}}^{+}↔{\left[{\mathrm{GAH}}_{x}\right]}_{\left(\mathrm{sol}\right)}^{x+}$$

where ${{[\mathrm{GAH}}_{x}]}_{\left(\mathrm{sol}\right)}^{x+}$ is the protonated inhibitor in the solution. As mentioned before, an increase in CO_2_ partial pressure leads to an increase in the acidity of the solution [32]. The higher value of *IE* observed at higher CO_2_ partial pressures can be ascribed to the higher concentration of H^+^ ions present in the solution, which in turn leads to an increase in the number of protonated inhibitor molecules that can be adsorbed on the metal surface. Moreover, Figure 2 also reveals that *IE* varies with the concentration of the inhibitor until the system reached a state (e.g., 1.0 g L^−1^ of GA), in which it can be said that the inhibitor molecules are in equilibrium with their protonated counterpart. For further increase in GA concentration, *IE* remains almost stable. The results clearly demonstrate that GA has greatly reduced the *CR* of the metal in the tested environment, and the high corrosion inhibition activity of GA was influenced by both its concentration and CO_2_ partial pressure. The lower values of *CR* observed in the presence of the inhibitor can be ascribed to its adsorption on the metal surface, covering the metal surface and thereby, blocking the active corrosion sites on its surface [4,7,28]. The gravimetric results are also supported by the SEM analysis presented from Figures 7–9, where it can be seen that the surface coverage increases and the protective layer becomes more compact in the presence of GA and with increasing CO_2_ partial pressure.

As the temperature rises, *IE* slightly decreased. This decrease may be due to the combination of two different reasons. For instance, the solubility of CO_2_ decreases with increasing the temperature of the solution [31], which can lead to a less acid environment. The pH of the solution increases slightly and therefore shifting the equilibrium reaction Equation (12) to the left. At higher pH, the concentration of H^+^ ions in the solution is smaller, which would result in the formation of fewer protonated inhibitor molecules available for the absorption process. Another possible reason may be due to the fact that these types of inhibitors get absorbed via electrostatic interactions (e.g., van der Waal forces) onto the surface of the metal, and it is known that this types of interaction generally grow weaker with an increase in temperature due to larger thermal motion [3,20]. Consequently, an increase in temperature will increase the metal surface kinetic energy, which has a detrimental effect on the adsorption process and encourages desorption processes [15,20].

Table S2 lists the inhibition efficiency of various corrosion inhibitors used to mitigate sweet corrosion obtained at different immersion times and temperatures. It is worth mentioning that most of these inhibitors are labeled either as toxic or are expensive to synthesize. Umoren et al. [15] reported the corrosion inhibition efficiency of a commercial inhibitor to be 87 and 88% at 25 and 60 °C, respectively after 24 h of immersion. The table shows that GA, compared to other studied corrosion inhibitors, and the commercial corrosion inhibitor, can be considered a good environmentally friendly corrosion inhibitor for carbon steel in a CO_2_-saturated saline solution. Moreover, since GA is already used as a thickening agent in the make-up of the fracturing fluid, can also work as an active component in corrosion inhibitor in the shale gas industry.

#### 3.1.2. Electrochemical Experiments

The electrochemical experiments such as electrochemical impedance spectroscopy (EIS) and potentiodynamic polarization (PDP) were also employed as a means to support the gravimetric findings. These experiments were carried out at 1.0 g L^−1^ of GA at different CO_2_ partial pressures after 24 of immersion. 1.0 g L^−1^ is the concentration in which the tested inhibitor exhibited a maximum in the concentration-efficiency curve.

**Figure 3:**
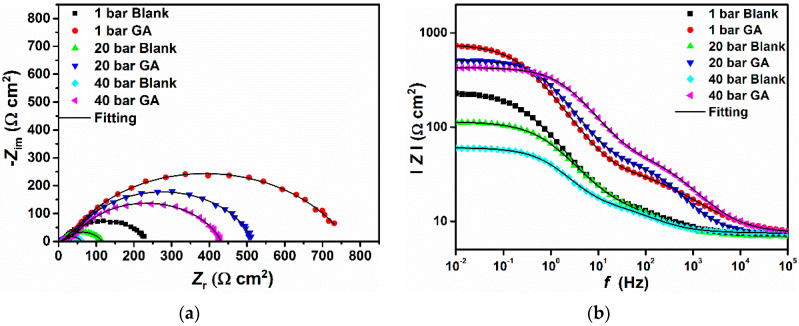

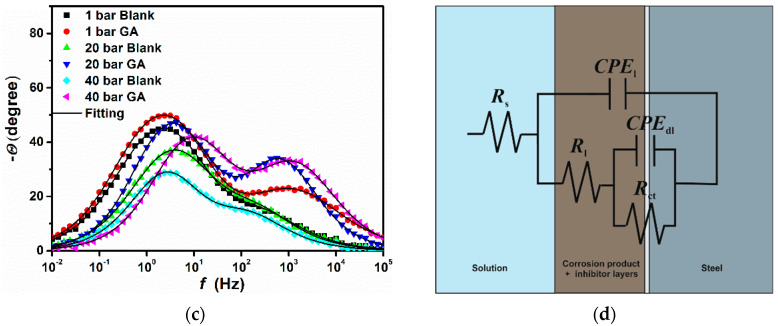
EIS plot recorded in the presence and absence of 1 g L^−1^ of GA after 24 h of immersion at different CO_2_-partial pressures. (**a**) Nyquist; (**b**) Bode; (**c**) phase angle; (**d**) equivalent circuit

The EIS measurements were used to evaluate the resistance of the protective layer from the electrochemical angle and are presented in Figure 3. It can be seen from the Bode (Figure 3b) and phase angle plots (Figure 3c) that the system is characterized by two-time constants at low (LF) and high frequencies (HF). The presence of these two-time constants suggests that the electrochemical reaction process of the N80 carbon steel in a CO_2_ saturated saline solution is affected by two state variables i.e., the corrosion products layer and/or the protective adsorptive layer, and electric double-layer, as also reported by Dong et al. [33]. For this reason, the EIS plots presented in Figure 3 were fitted with the help of the equivalent circuit (EC) presented in Figure 3d, consisting of the following elements: *R*_s_ is the electrolyte resistance. *CPE*_l_ and *R*_l_ are the constant phase element and the resistance of the layer formed on the metals surface, respectively. *CPE*_dl_ and *R*_ct_ are the constant phase element representing the double-charge layer capacitance and the charge transfer resistance, respectively. The EIS parameters are listed in Table 1 and from the small values of *χ*^2^ (i.e., the goodness of fit) it can be said that the EC used to fit the system under investigation was the most appropriate one.

The presence of a time constant at HF is reported in several studies [34,35] and it is often observed in a Fe/water system. This time constant may be due to the capacity of a porous thin layer formed onto the metal surface. In this study, and without the inhibitor, the presence of this time constant at HF is due to the formation of a thin layer of Fe_3_C onto the metal surface. As mentioned before, the microstructure of the tested carbon steel is composed of circa 41% of a ferritic phase and the remaining of a perlitic phase (Figure 1). The ferritic phase is more active than the Fe_3_C contained in the perlitic phase [7], in this case, the former phase will act as an anode and the latter one as a cathode. This will generate a micro-galvanic effect, which will eventually lead to the formation of a thin layer of Fe_3_C onto the metal surface. However, it follows from the data that both the values of *R*_f_ and *R*_ct_ greatly increased in the presence of the inhibitor, which indicated that the GA molecules were adsorbed onto the metal surface leading to the formation of a protective layer that covers the surface, as confirmed also from the morphological analysis (e.g., SEM-EDS and XPS). Moreover, the difference between these two values obtained in the absence and the presence of GA increased even more with increasing CO_2_ partial pressure, suggesting that this protective layer becomes more stable and compact, with the corrosion inhibition efficiency going from 69.83% up to 87.44% at $P_{{\mathrm{CO}}_{2}}$ = 1 bar and $P_{{\mathrm{CO}}_{2}}$ = 40 bar, respectively. The increase in *IE* observed with an increase in CO_2_ partial pressure agrees with the results obtained with the gravimetric measurements and is in agreement with the ones reported in the literature [4]. It is evident that the addition of GA had a remarkable effect on the corrosion process of the metal and that its inhibition not only depends on the concentration of GA but also from CO_2_ partial pressure. The results show that the coverage and thickness of the formed protective layer increased with CO_2_ partial pressure, acting both as a barrier against the charge and the mass transfer processes that occur onto the metal surface owing to the corrosive attack of the aggressive electrolyte.

**Figure 4:**
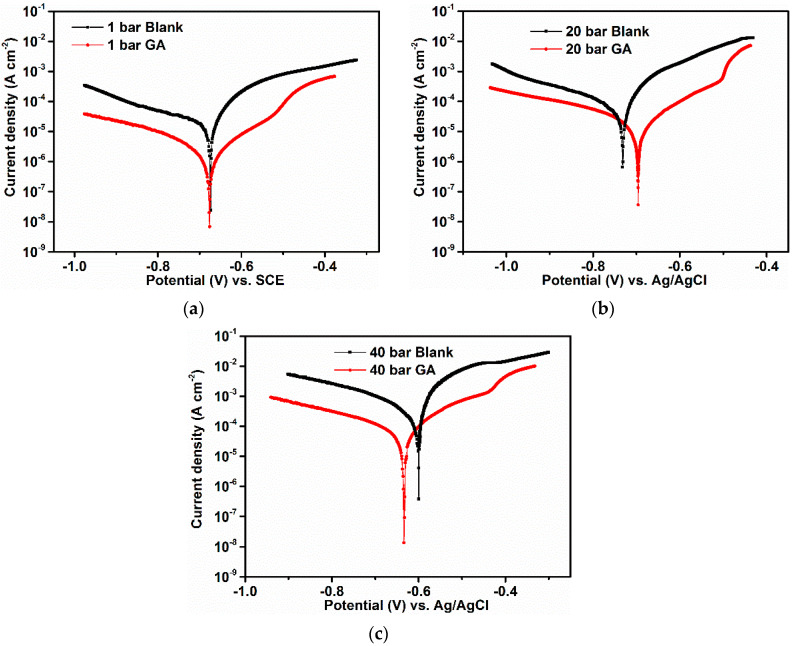
Potentiodynamic polarization parameters obtained in the absence and presence of 1.0 g L^−1^ of GA at different CO_2_-partial pressures, after 24 h of immersion. (**a**) $P_{{\mathrm{CO}}_{2}}$ = 1 bar, (**b**) $P_{{\mathrm{CO}}_{2}}$ = 20 bar and (**c**) $P_{{\mathrm{CO}}_{2}}$ = 40 bar.

Figure 4 and Table 2 show the potentiodynamic polarization measurements and the corrosion kinetic parameters obtained from the polarization plots in the presence and absence of GA at different CO_2_ partial pressures, respectively. As can be seen from Figure 4, the anodic polarization curve of the blank solution does not show the typical Tafel behavior consequently, the corrosion current densities were calculated from the extrapolation of the cathodic Tafel region.

The data shows that in absence of GA, the corrosion current density of the steel increased with an increase in CO_2_ partial pressure, which is linked with the increased acidity of the solution, in agreement with the gravimetric experiments. However, it is evident from the data that the corrosion current density of the steel was prominently reduced after the addition of GA to the solution. Furthermore, both the cathodic and anodic curves of the polarization curves were shifted towards lower current densities after the addition of GA. The result suggests that the inhibitor impeded both the rate of the anodic dissolution (Equation (8)) and the cathodic reactions (Equations (9)–(11)), by either covering part of the metal surface and/or blocking the active corrosion sites on the steel surface. The dominant cathodic reaction depends on the pH value of the solution. At lower pH (e.g., less than 4) the reduction of H^+^ ions would be the dominant cathodic reaction (Equation (11)). At pH > 4 the dominant cathodic reaction will be the reduction of ${\mathrm{HCO}}_{3}^{-}$ ions and H_2_CO_3_ (Equations (9) and (10)). At higher values of CO_2_ partial pressure, GA suppresses the Equation (11) (e.g., the pH of the solution is circa 3.5 at $P_{{\mathrm{CO}}_{2}}$ = 40 bar), through the formation of H-bonding between the hydroxyl groups of the inhibitor units and the H^+^ ions, adsorbed onto the steel surface, as discussed in more detail in Section 3.5.2.

Moreover, after the addition of GA, the *E*_corr_ can be seen to shift with no definite trend toward both the anodic and cathodic regions. This result suggested that GA behaves as a mixed type inhibitor as also reported by other studies for this inhibitor [7,21,27,28].

### 3.2. Effect of Time

**Figure 5:**
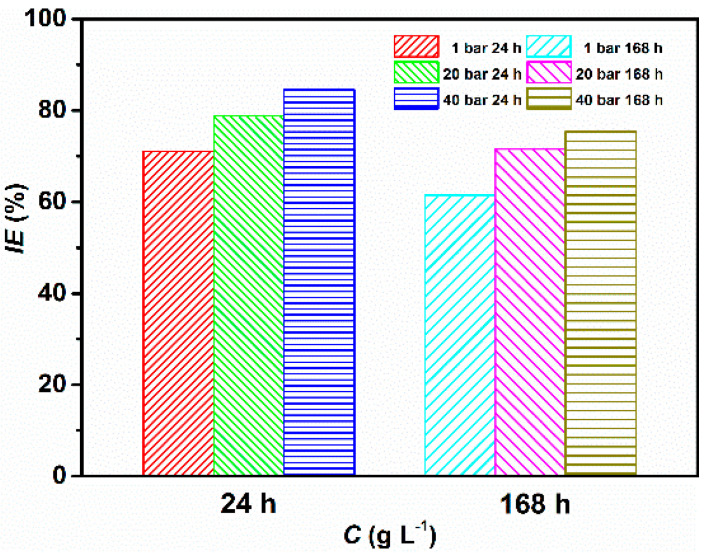
Corrosion inhibitor efficiency obtained at different CO_2_ partial pressures after 24 and 168 h of immersion at 25 °C.

The effect of immersion time on the corrosion inhibition efficiency of the tested inhibitor was also assessed in this paper. Figure 5 and Table S3 show the corrosion rate and the corrosion inhibition efficiency after 168 h of immersion in the presence and absence of 1.0 g L^−1^ of GA at different CO_2_ partial pressures at 25 °C. It follows from the table that GA still shows a very high *IE* even after a longer immersion time. However, it should be noted that *IE* slightly decreases after 168 h of immersion, compared to the one observed after 24 h of immersion.

This behavior has also been reported by several studies [36,37]. The decrease in *IE* may be due to the desorption of the inhibitor from the metal surface, which makes the protective layer unstable. In this study, the desorption of GA is likely ascribed to its deprotonation due to the consumption of CO_2_ from the tested solution because of the electrochemical reactions occurring into the system [5,38]. This leads to a decrease in the acidity of the solution and shifting the Equation (12) towards the deprotonation of the inhibitor.

These results confirm that GA is effectively able to protect the steel surface from sweet corrosion at high CO_2_ partial pressures even after a prolonged immersion time, reflecting a strong molecular adsorption of GA on the metal surface and the formation of a stable protective layer.

### 3.3. Adsorption Study and Standard Adsorption Free Energy

The corrosion inhibition adsorption process of the tested inhibitor on the N80 carbon steel surface was carried out by several adsorption isotherms, such as Temkin’s, Frumkin’s, Langmuir’s, and El-Awady’s adsorption isotherms. The Temkin’s adsorption isotherm was found to give the best description of the adsorption behavior of the studied inhibitor. The Temkin’s adsorption isotherm is defined by the following equations:

(13) $$\theta =\frac{-2.303\;\log K_{\mathrm{ads}}}{2a}-\frac{2.303\;\log C}{2a}\;$$

where *θ* is the surface coverage (*θ* = *IE*%/100), *K*_ads_ the adsorption-desorption equilibrium constant, *C* is the inhibitor concentration, *a* is the molecules interaction parameter. Positive values of *a* imply attractive forces between the inhibitor molecules, while negative values indicate repulsive forces between them.

**Figure 6:**
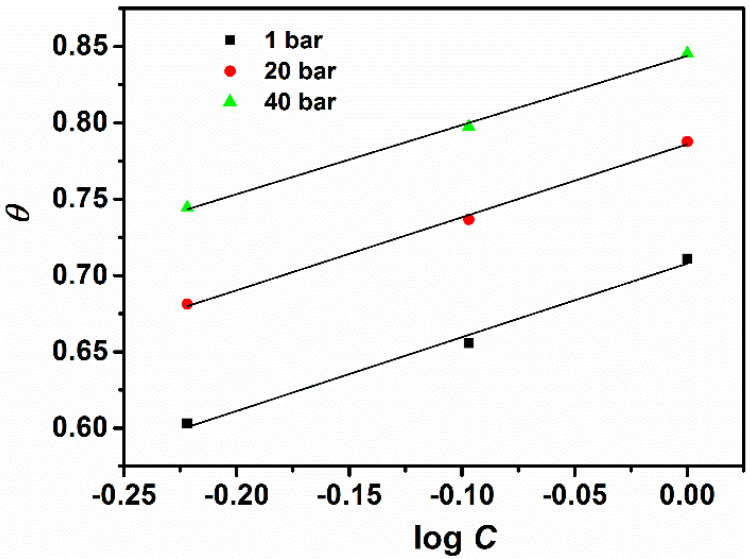
Temkin’s adsorption isotherm for carbon steel (N80) pipeline steel in CO_2_-saturated chloride at different pressures.

*K_ads_* is related to the free energy of adsorption by the following equation:

(14) $$\Delta G_{\mathrm{ads}}^{{}^{\circ}}=-{RT\;\mathrm{Ln}(K}_{\mathrm{ads}})$$

where *R* is the gas constant (8.314 J K^−1^ mol^−1^), *T* is the absolute temperature (K). The plot of surface coverage (*θ*) as a function of the logarithm of the inhibitor concentration at different CO_2_ partial pressures is shown in Figure 6.

The plot of *θ* vs. log *C* yields a straight line and the regression coefficient ranges from 0.985 to 0.996. The calculated values of adsorption parameters Δ*G*°_ads_, *a* and *K* at different CO_2_ partial pressures are presented in Table 3 and the following notes can be written: (i) The values of Δ*G*°_ads_ are negative for all three pressures, indicating that the adsorption of GA on the steel surface in the tested solution is a spontaneous process [7,21,22,28]. Furthermore, the value of Δ*G*°_ads_ ranges between −10.64 to −8.37 kJ mol^−1^ indicating that the adsorption of GA on the steel occurs through a physical adsorption process [7,21,22,28]; (ii) The values of “*a*” are negative for all three pressures, indicating that repulsion forces exist between the adsorbed inhibitor molecules in the adsorption layer, as also reported by other studies for the same tested inhibitor [7,22]; (iii) The values of *K*_ads_ increases with an increase in CO_2_ partial pressure. It should be noted that *K*_ads_ denotes the strength between adsorbate and adsorbent. It can be inferred that a large value of *K*_ads_ implies a more efficient adsorption process and thus, a better corrosion inhibition efficiency [21,22]. The results suggest that the adsorption of GA increases with an increase of the environment pressure, leading to a greater surface coverage and consequently, a better protection performance.

### 3.4. Surface Analysis

**Figure 7:**
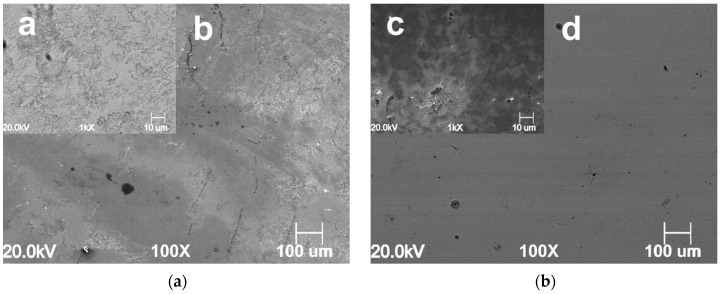
SEM images of the N80 carbon steel surface morphology after 24 h of immersion in the uninhibited ((**a**) **a** lower and **b** higher magnification) and inhibited ((**b**) **c** lower and **d** higher magnification) solution at 25 °C and $P_{{\mathrm{CO}}_{2}}$ = 1 bar CO_2_.

**Figure 8:**
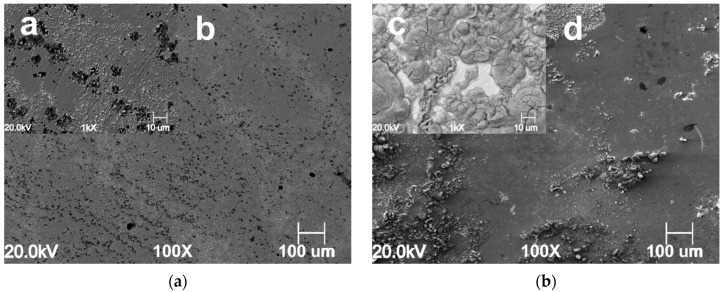
SEM images of the N80 carbon steel surface morphology after 24 h of immersion in the uninhibited ((**a**) **a** lower and **b** higher magnification) and inhibited ((**b**) **c** lower and **d** higher magnification) solution at 25 °C and $P_{{\mathrm{CO}}_{2}}$ = 20 bar CO_2_.

**Figure 9:**
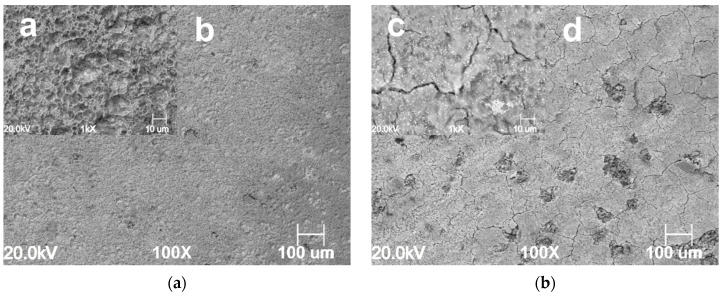
SEM images of the N80 carbon steel surface morphology after 24 h of immersion in the uninhibited ((**a**) **a** lower and **b** higher magnification) and inhibited ((**b**) **c** lower and **d** higher magnification) solution at 25 °C and $P_{{\mathrm{CO}}_{2}}$ = 40 bar CO_2_.

The surface morphology of the samples exposed for 24 h at different CO_2_ partial pressures in the absence and presence of 1.0 g L^−1^ of GA are presented in Figure 7, Figure 8 and Figure 9. For instance, it can be seen that the surface morphology of the samples exposed to the blank and inhibited solution differs significantly. For the blank solution, at $P_{{\mathrm{CO}}_{2}}$ = 1 bar, the microstructure of the sample is clearly visible (Figure 7a). The metal surface appears corroded resulting from the selective dissolution of the ferritic phase over the cementite contained in the perlitic phase. By contrast, Figure 7c,d show that after the addition of the inhibitor the metal surface becomes much smoother. It is clear from the image that the metal surface was partially covered by a protective layer, although some areas of the surface still show signs of corrosion attacks.

The severity of the corrosion attack increases with an increase in CO_2_ partial pressure in the blank solution, as shown in Figure 8a,b and Figure 9a,b respectively carried out at $P_{{\mathrm{CO}}_{2}}$ = 20 bar and $P_{{\mathrm{CO}}_{2}}$ = 40 bar. However, it can be seen that in the presence of GA, an increase in CO_2_ partial pressure led to a gradual increase in the surface coverage on the metal surface, as a result of an increase of GA molecules adsorbed onto the metal surface (Figure 8c,d and Figure 9c,d). At higher CO_2_ partial pressure (e.g., $P_{{\mathrm{CO}}_{2}}$ = 40 bar, Figure 9) the protective action of the inhibitor is even more evident. The images show that for the uninhibited solution, the surface of the metal appears severely corroded, while the one obtained in the presence of the inhibitor shows the formation of a uniform protective layer over its entire metal surface. The results indicate that in the presence of GA and with a gradual increase in CO_2_ partial pressure, the protective layer gradually becomes more compact and thicker [4]. As discussed in Section 3.1, the solubility of CO_2_ increases with its partial pressure, and as a result of this, the concentration of H^+^ ions into the solution also increases, hence the number of the inhibitor molecules that can be protonated and adsorbed onto the metal surface also increases according to the Equation (12), leading to a substantial reduction of the corrosion rate of the metal.

**Figure 10:**
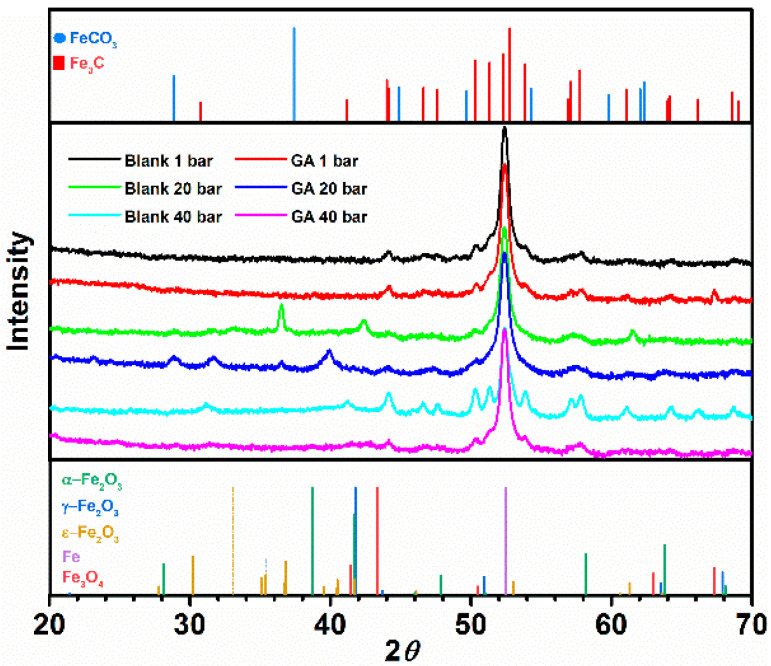
XRD spectra of corrosion product film formed on the metal surface after been exposed for 24 h without and with the presence of 1.0 g L^−1^ of GA at different CO_2_ partial pressures at 25 °C.

The morphology of the metal surface was also analyzed with the help of an energy-dispersive spectroscopy with the result listed in Table 4. In the absence of GA, the metal surface was characterized by a corrosion product layer mainly consisting of carbon, iron, and a small amount of oxygen elements, indicating that this corrosion layer is mainly composed of Fe_3_C. These results are in agreement with that previously observed in the literature [5,7,39,40]. Other researchers reported that at a temperature below 40 °C, the corrosion product layer is generally composed of Fe_3_C, and only little traces of FeCO_3_ were observed on the metal surface [4,7,39,40], as also confirmed by the GIXRD measurements shown in Figure 10. The presence of Fe_3_C on the metal surface is due to the anodic dissolution of the ferrite phase over the cementite in the perlitic phase, which leads to an accumulation of the cementite on the metal surface.

It is worth mentioning that in the presence of GA the content of carbon and oxygen was found to be higher than those observed for the blank solution. It should be noted that carbon and oxygen are also the main constituents of the tested inhibitor and therefore, their higher concentration on the protective layer formed in the presence of the inhibitor can be attributed to its adsorption onto the metal surface, as also reported by other studies [4,7,20]. Moreover, it can be seen from the table that the percentage of Fe decreased in the presence of GA, likely due to the overlying effect of the inhibitor layer.

The GIXRD analysis for the samples corroded in an inhibited and uninhibited solution at $P_{{\mathrm{CO}}_{2}}$ = 40 bar and at 25 °C (Figure 10) shows the presence of cementite on the metal surface, although in the presence of GA the intensity of these peaks is much weaker. This result can be explained as follows: Fe_3_C accumulates on the metal surface after the dissolution of the ferritic phase. However, in the presence of the inhibitor, it only accumulates in small amounts on the bare metal surface at the early stage of the experiment, since the dissolution of the ferritic phase is quickly suppressed by the absorption of the inhibitor on the surface of the metal.

**Figure 11:**
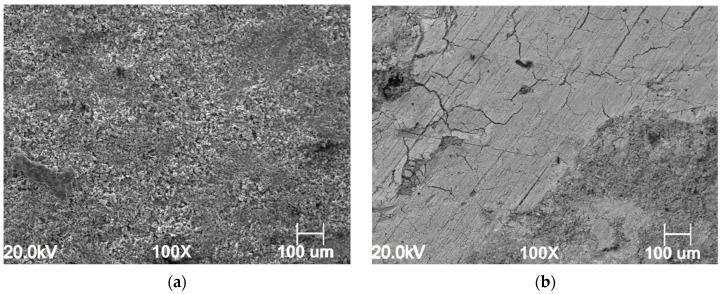
SEM images of the N80 carbon steel morphology after 24 h of immersion in the tested solution at $P_{{\mathrm{CO}}_{2}}$ = 40 bar and at 60 °C, without (**a**) and with (**b**) the presence of GA.

Figure 11a,b show the surface morphology for specimens corroded in the blank and inhibited solution carried out at 60 °C and $P_{{\mathrm{CO}}_{2}}$ = 40 bar, after immersion the samples for 24 h in the tested solution, without and with the presence of GA, respectively. The corrosion product layer appears to be different for the inhibited solution compared to one observed in the presence of GA. Figure 11a shows the presence of a porous corrosion product layer formed onto the metal surface corroded in a free-inhibitor solution, pores which create paths for the solution to penetrate it and thereby leading to the dissolution of the underlying metal. On the other hand, the surface of the metal corroded in the presence of GA (Figure 11b) shows the formation of a more compact layer, which forms a better protective barrier and thereby greatly reducing the corrosion rate of the metal.

**Figure 12:**
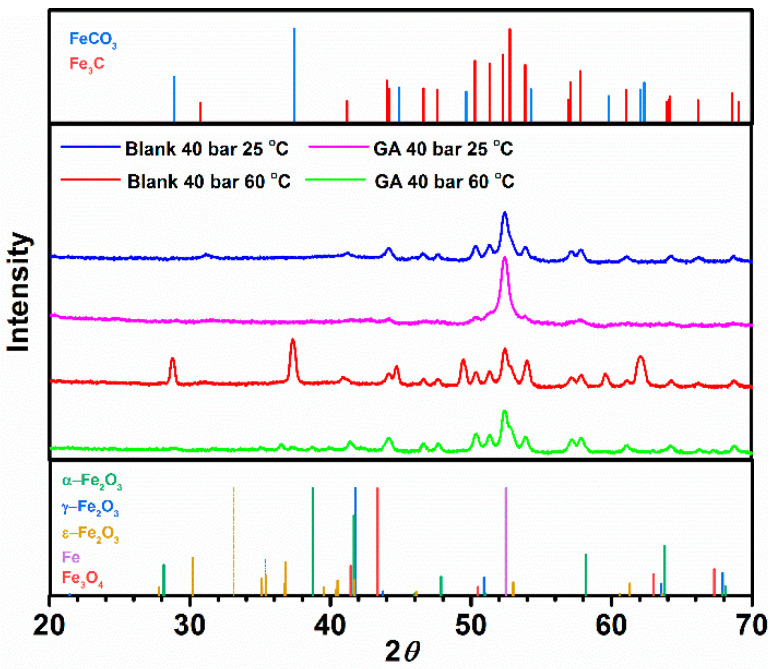
XRD spectra of corrosion product film formed on the metal surface after been exposed for 24 h without and with the presence of 1.0 g L^−1^ of GA at $P_{{\mathrm{CO}}_{2}}$ = 40 bar and at 60 °C.

EDS analysis reports high content of carbon, oxygen, and iron elements in both layers (C:11.11%, O:6.02% and C:13.69%, O:11.0%, in the blank and inhibited solution, respectively). The GIXRD measurements presented in Figure 12 show the characteristic XRD diffraction patterns associated with FeCO_3_. By contrast, the intensity of the iron carbonate peaks observed in the presence of GA is almost negligible. These results suggest that the layer observed for the uninhibited solution is mainly composed of Fe_3_C and FeCO_3_, while in the presence of GA is mainly composed of Fe_3_C with little traces of FeCO_3_ [4]. Similar behavior was also reported by Ding et al. [41] related to the study of the effect of an imidazoline-type inhibitor against CO_2_ corrosion of mild steel. The authors suggested that the formation of the corrosion inhibitor layer was able to suppress the formation of the iron carbonate. The precipitation of FeCO_3_ depends on the concentration of the Fe^2+^ and ${\mathrm{CO}}_{3}^{2-}$ ions, pH, and temperature. When the concentrations of Fe^2+^ and ${\mathrm{CO}}_{3}^{2-}$ ions exceed the solubility limit, FeCO_3_ will precipitate on the surface [9,40,42]. At higher temperatures, its solubility decreases, and therefore the likelihood of its precipitation will be also higher. In a free-inhibitor solution, the dissolution of the ferrite phase may lead to an increase in the concentration of Fe^2+^ ions in the bulk solution and thereby favoring the precipitation of FeCO_3_ onto the surface of the metal. Conversely, in the presence of the inhibitor, the protective layer formed onto the surface of the metal slows down the corrosion processes, and thereby reducing the concentrations of Fe^2+^ ions available for the formation of FeCO_3_.

**Figure 13:**
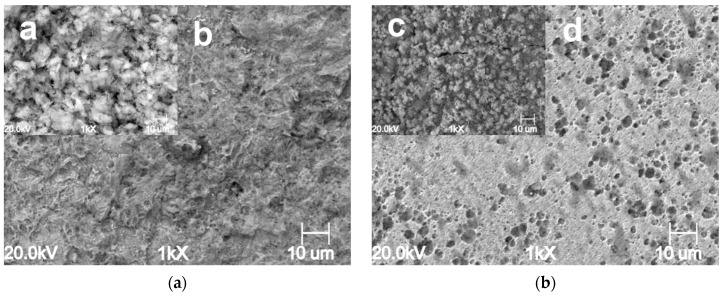
SEM images of the N80 carbon steel morphology after 168 h of immersion in the tested solution in the presence of 1.0 g L^−1^ of GA at $P_{{\mathrm{CO}}_{2}}$ = 40 bar. Without (**a**,**b**) and with the inhibitor (**c**,**d**) at 25 °C.

Figure 13a–d show the SEM analysis of the metal surface after 168 h of immersion in the absence and presence of 1.0 g L^−1^ GA at $P_{{\mathrm{CO}}_{2}}$ = 40 bar, respectively. It is apparent from the figures that a thick porous layer covers both surface samples; although it seems that in the presence of the GA, this layer appears denser, thus providing a higher level of protection. To analyze the condition of the metal surface, these porous layers were removed with the help of Clark’s solution. It can be seen that both surfaces show clear signs of corrosion attacks (Figure 13b; however, it is also clear from the figures that in the presence of the inhibitor (Figure 13d) the surface of the metal appears to be less damaged and smoother, with the ground scratches still visible on the surface. This result was also confirmed by the atomic force microscopy experiments performed by Azzaoui et al. [28] concerning the use of GA as a corrosion inhibitor in a 1 M HCl solution. The authors reported that in the uninhibited solution the surface of the metal was found to be more corroded with an average roughness of 1.3 μm, while in the presence of GA the average roughness was reduced to 500 nm. The authors justified this behavior due to the formation of a more compact protective layer on the metal surface that strongly reduced the diffusion of the aggressive substances to the metal, and thereby reducing the corrosion rate of the metal.

The SEM-EDS and GIXRD result confirm that GA provides adequate protection to the metal surface from sweet corrosion even at high CO_2_ partial pressures and after long immersion times. The results are in agreement with the findings obtained with the weight loss measurements, confirming the high inhibition efficiency value observed after a long immersion time.

**Figure 14:**
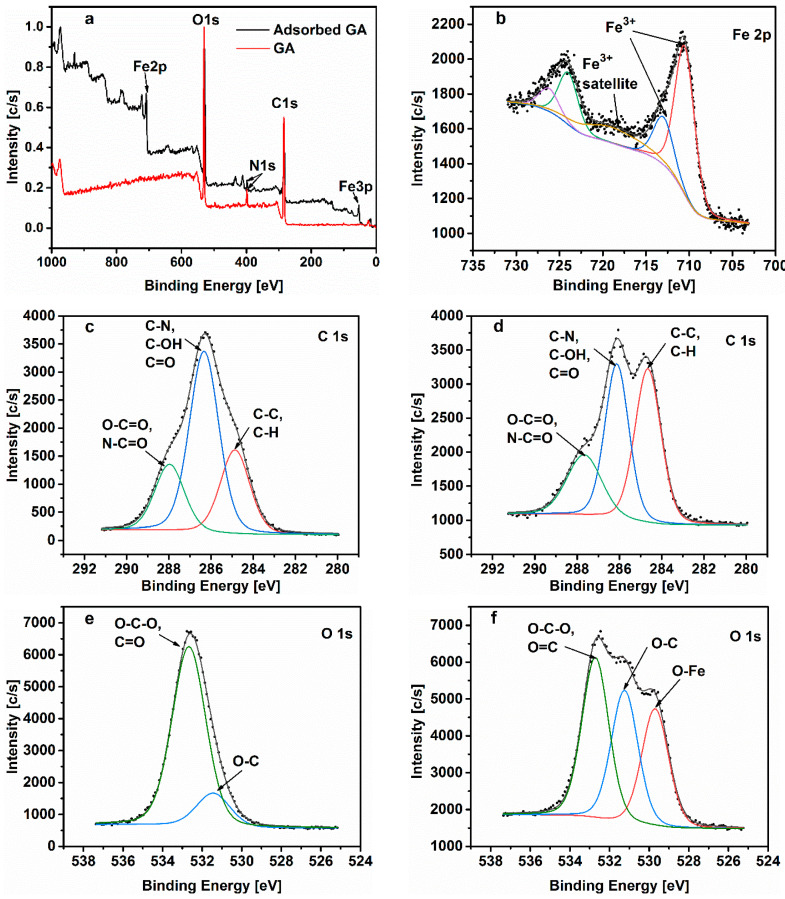

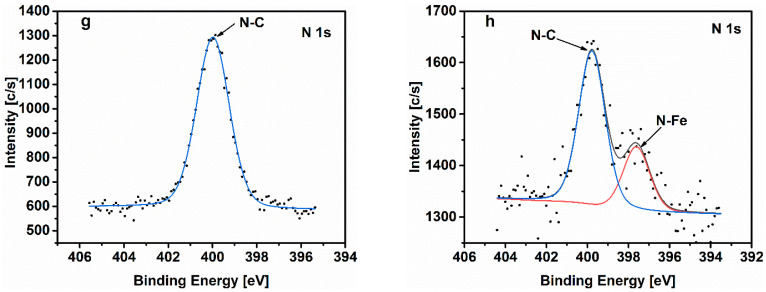
XPS spectra of the native gum Arabic: (**a**,**c**,**e**,**g**). XPS spectra of the film formed on the N80 carbon steel after 24 h exposure in CO_2_ at $P_{{\mathrm{CO}}_{2}}$ = 40 bar in the presence of 1.0 g L^−1^ of GA at 25 °C: (**b**,**d**,**f**,**h**).

X-ray photoelectron spectroscopy analysis was employed as a means to confirm the adsorption of the tested inhibitor on the carbon steel surface. The analysis was carried out on the native inhibitor and the steel surface after 24 h of immersion in the tested solution in the presence of 1.0 g L^−1^ of GA at $P_{{\mathrm{CO}}_{2}}$ = 40 bar and at 25 °C. The XPS results presented in Figure 14a showed evidence of the presence of O, C, N, and Fe on the carbon steel surface, where the O and C contents displayed the highest amount, while the signal of N was detected with small intensity. The high-resolution peaks core levels were analyzed through a deconvolution fitting of the complex spectra. The binding energies and the corresponding quantification (%) of each peak component are presented in Table S4.

The deconvoluted Fe2p_3/2_ peaks (Figure 14b) at 710.5 and 713.0 eV could be associated with the α-Fe_2_O_3_ or/and γ- Fe_2_O_3_ [13]. The presence of these species is likely due to the partial decomposition of iron carbonate. The literature reported that FeCO_3_ begins decomposing at temperatures below 100 °C according to the following reaction [5,42]:

(15) $${\mathrm{FeCO}}_{3}\to \mathrm{FeO}+{\mathrm{CO}}_{2}$$

In the presence of CO_2_ or water vapor, FeO transforms into Fe_3_O_4_ [5,42].

(16) $$3\mathrm{FeO}+{\mathrm{CO}}_{2}\to {\mathrm{Fe}}_{3}O_{4}+\mathrm{CO}$$

(17) $$3\mathrm{FeO}+H_{2}O\to {\mathrm{Fe}}_{3}O_{4}+H_{2}$$

However, in the presence of oxygen, FeO and Fe_3_O_4_ transform into Fe_2_O_3_ [5,42].

(18) $$4\mathrm{FeO}+O_{2}\to {2\mathrm{Fe}}_{2}O_{3}$$

(19) $$\mathrm{In}\;\mathrm{the}\;\mathrm{air}:\;4{\mathrm{Fe}}_{3}O_{4}+O_{2}\to {6\mathrm{Fe}}_{2}O_{3}$$

**Figure 15:**
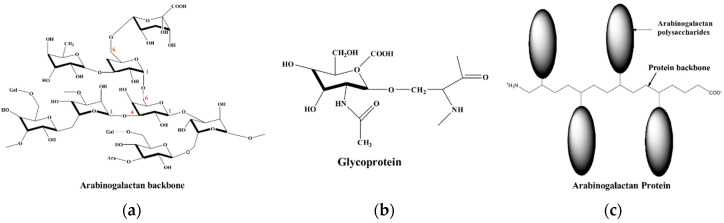
Structure of gum arabic: (**a**) arabinogalactan; (**b**) glycoprotein; (**c**) arabinogalactan-protein.

The C1s spectra of the native gum arabic and the adsorbed one (Figure 14c,d, respectively) show three main peaks. The C1s peak with binding energy at 284.8 eV could be attributed to the C–C/C–H bonds [26,28]. The C1s peak at 286.2 eV could be attributed to the C–OH/C=O bonds related to the different groups of GA [26,43]. This peak may also be assigned to the carbon atom bonded to nitrogen in C–N bond [13,44] and could be related to the glycoprotein and/or to the arabinogalactan-protein fractions of the inhibitor (Figure 15b,c, respectively). The last C1s peak with a binding energy of 287.7 eV could be associated with the presence of carbonyl type groups O–C=O/N–C=O that result from the protonation of the GA molecule in the acid environment [28].

It is worth mentioning that no peaks assigned to Fe_3_C were found with the XPS analysis in contrast to the results reported from the GIXRD analysis, where the characteristic peaks assigned to this compound can be seen in the presence of GA (Figure 10). Fe_3_C cannot be detected since the average depth of analysis for an XPS measurement is approximately 5 nm however, the cementite formed on the metal surface at the early stage of the experiment is covered by a thicker layer of inhibitor (Figure 9c,d).

The deconvoluted O1s spectra of the native and adsorbed inhibitor are displayed in Figure 14e,f, respectively. The peaks at 531.2 and 532.7 eV could be attributed to the single bonded oxygen in C–O and the double bonded oxygen C=O and/or to the single bonded oxygen in O–C–O respectively [4,13,26,28]. The latter peak may correspond to the carbonyl type groups and/or to the glycosidic C(1)-O-C(4)/C(1)-O-C(6) linkages of the GA molecules (Figure 15a), as well as, in the case of the sample exposed to the tested solution, to FeCO_3_ formed on the metals surface, respectively [4,26,28]. Moreover, some authors reported that the peak at 231.2 eV could also be attributed to the oxygen of the hydroxyl groups (–OH) [5,43], likely due to the hydroxyl groups of the tested polysaccharide. The O1s spectrum of the adsorbed inhibitor (Figure 14f) displays an extra peak at 529.7 eV corresponding to O^2−^ related to the oxygen atoms bonded with Fe^3+^ in the Fe_2_O_3_ oxide [4,43,44]. The O1s results are in good agreement with the findings of the Fe2p spectrum.

The presence of N1s peak in the survey for the adsorbed GA on the carbon steel surface (Figure 14a) provides evidence that gum arabic was effectively adsorbed on the tested substrate surface since the N80 carbon steel substrate does not contain nitrogen in its chemical composition. The N1s spectra of the native and adsorbed inhibitor are presented in Figure 14g,h. Both images show the presence of a peak at 400 and 399.8 eV attributed to the nitrogen atom bonded with the carbon atom, for the native and adsorbed inhibitor. However, as it can be seen that the high-resolution N1s spectrum of the tested substrate sample after the addition of GA depicts an extra peak at 397.6 eV. This extra peak can be ascribed to the coordinated nitrogen atom of the amino group with the metal surface (N–Fe bond) [44]. Other authors also suggested that this peak could be attributed to the bond between the nitrogen of the amino groups and the oxide layer on the metal surface (FeO_x_) [45].

### 3.5. Mechanism of Inhibition

Given all the observed results, it can be inferred that the GA was effectively adsorbed on the metal surface, providing good protection to the metal surface against sweet corrosion. However, the complex chemical structure of this inhibitor makes it difficult to determine the exact adsorption mechanism involved. Gum arabic is a heterogeneous mixture of different compounds consisting of three main fractions: 80% of arabinogalactan (AG), 10.4% of arabinogalactan-protein (AGP) and 1.2% glycoprotein (GP) (Figure 15). Each of these fractions contains a range of different molecular weight components and different protein contents. Therefore, some of these compounds can be physically and others chemically adsorbed. Nevertheless, based on the results reported in this study, it can be assumed that the following three types of adsorption mechanisms or likely a combination of them may take place in the inhibiting phenomena involving GA on the steel surface.

#### 3.5.1. Adsorption via Electrostatic Interaction

**Figure 16:**
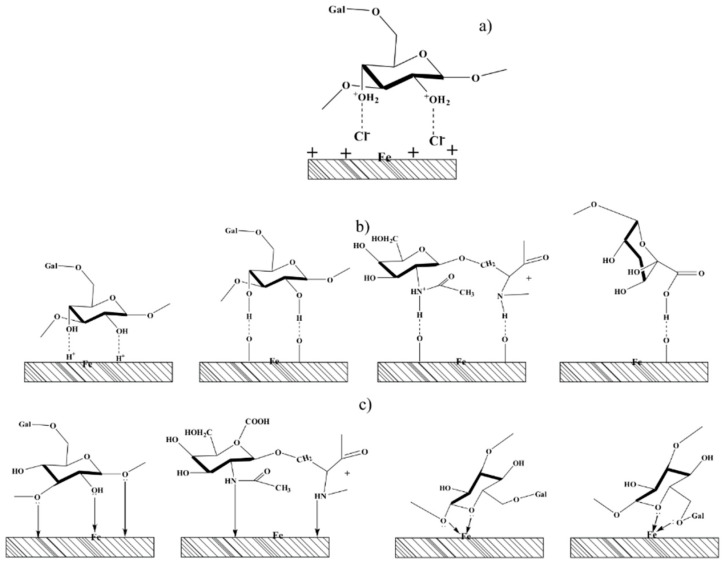
Schematic representation of the corrosion inhibition mechanism of the N80 carbon steel by GA. (**a**) electrostatic; (**b**) H-bond formation; (**c**) chemical adsorption.

The functional groups such as hydroxyl, carboxyl, and amino present in the GA molecules, by virtue of the presence of lone pair of electrons, can be easily protonated in acid solutions such that the newly formed polycations are in equilibrium with their neutral counterpart according to the Equation (12). The high corrosion inhibition activity showed by GA is likely due to a synergistic electrostatic interaction between the protonated GA molecules with the adsorbed chloride ions, as shown in Figure 16a. As reported by several studies [7,20,21,22,28] chloride ions are strongly adsorbed on the positively charged metal surface, thereby creating an excess of electrons so that the metal will be negatively charged. These adsorbed chloride ions can act as an intermediate bridge between the surface and the protonated inhibitor molecules and therefore, assisting the adsorption of GA on the metal surface. This type of adsorption mechanism is likely the one that accounts for the most inhibition action of the inhibitor. In fact, the results presented in this manuscript have demonstrated clearly that the corrosion inhibition action of GA was strongly influenced by both the concentration of the inhibitor, CO_2_ partial pressure, and temperature. A change in one of these two factors has a great effect on the equilibrium reaction (Equation (12)), shifting the equilibrium towards the protonated or the deprotonated form of the inhibitor. A shift to the right implies an increase in the number of protonated molecules of GA available to interact with the chloride ions adsorbed on the surface and thus, an increase in *IE* of the system.

#### 3.5.2. Adsorption via Hydrogen Bond Formation Interaction

At higher CO_2_ partial pressure (i.e., 40 bar) the pH of the solution is around 3 [32], and among the three possible occurring cathodic reactions (Equations (9)–(11)), the reduction of hydrogen ions to hydrogen gas is the dominant cathodic reaction. It is generally accepted that this reaction can be described using three steps [46]. The first step is the electrochemical adsorption of the H^+^ ions (Equation (20)) followed by either the electrochemical desorption (Equation (21)) or the chemical desorption (Equation (22)).

(20) $$H_{\left(\mathrm{aq}\right)}^{+}+e^{-}\to H_{\mathrm{ads}}$$

(21) $$H_{\mathrm{ads}}+H_{\left(\mathrm{aq}\right)}^{+}\to H_{2(g)}$$

(22) $$H_{\mathrm{ads}}+H_{\mathrm{ads}}\to H_{2(g)}$$

The potentiodynamic measurements presented in Figure 5 showed that the cathodic current density of the system was greatly reduced after the addition of GA in the solution, suggesting that GA was able to suppress the hydrogen evolution reaction (Equation (11)) to some extent. Similar results were also confirmed by other authors [21,26,27,28]. This assumption was also confirmed by FT-IR and Raman measurements performed on GA [7,26] and other gum-like [20,36,47] compounds. The results showed that the characteristic peak assigned to the hydroxyl groups of the carbohydrate units narrowed down and/or shifted after its adsorption on the metal surface. The authors agreed that this change in shape was likely due to a possible interaction of the hydroxyl groups of the GA molecules with the H adsorbed on the cathodic sites of the metal surface via H-bond formation (Figure 16b). Therefore, the high value of IE observed in this study at different CO_2_ partial pressure can be also ascribed to the ability of GA to suppress one of these reactions (Equations (20)–(22)) via H-bonds formation, thus suppressing Equation (11) and consequently the dissolution of the steel (Equation (8)).

The adsorption of GA may also be promoted by the presence of the oxide layer on the metal surface via hydrogen bonding (Figure 16b). Studies concerning the adsorption of GA on oxide nanoparticles (i.e., iron oxide nanoparticles [48] and zinc or aluminum oxide nanoparticles [49]) reported that GA showed a strong affinity toward these oxide nanoparticles. The authors suggested that the adsorption of GA on these oxide nanoparticles surface might be due to the formation of hydrogen bonds between the functional groups of the GA molecules (e.g., hydroxyl, carboxylate, and amino) with the oxidized surface. The XPS analysis presented in this study showed that the metal surface after 24 h of exposure is covered by different oxide species such as Fe_2_O_3_ and/or Fe_3_O_4_, (e.g., Equations (15)–(19)). Therefore, the adsorption of GA assisted by the presence of oxide species formed on the metal surface via H-bonds formation is an adsorption mechanism that must be also taken into account.

#### 3.5.3. Chemical Adsorption

The heteroatoms (i.e., O, N) present on the GA molecules by virtue of the presence of lone pair of electrons may promote the adsorption of the inhibitor via the formation of coordinate bonds with the iron from the metal surface and/or with iron from the oxide species formed on the surface [45] (Figure 16c). The XPS measurements observed in this study showed a peak at 397.6 eV likely ascribed to the coordinated nitrogen atom of the amino group with the Fe (N–Fe bond) [44]. This result suggests that although the inhibitor is mainly physically adsorbed on the surface of the metal, a small contribution of the chemical adsorption process cannot be ignored.

## 4. Conclusions

The corrosion inhibition effect of gum Arabic on the corrosion of carbon steel (N80) exposed in a high-pressure CO_2_-saline environment has been studied and the following conclusion can be drawn:

- The weight loss results showed that the thickening agent gum arabic was found to be an efficient corrosion inhibitor for carbon steel in a high-pressure CO_2_-saline environment. The Inhibition efficiency increased with an increase in inhibitor concentration and CO_2_ partial pressure with the maximum value of IE found to be 84.53% at $P_{{\mathrm{CO}}_{2}}$ = 40 bar after 24 h of immersion. Moreover, the weight loss results also showed that GA was effectively able to protect the steel surface from sweet corrosion at high CO_2_ partial pressures (i.e., 40 bar) even after a prolonged immersion time (i.e., 168 h) with a corrosion inhibition efficiency found to be 74.41%.
- The adsorption of GA on the carbon steel surface follows the Temkin’s adsorption isotherm model. The negative free energy of adsorption Δ*G*°_ads_ indicates a strong and spontaneous adsorption of GA on the carbon steel surface. Furthermore, the value of Δ*G*°_ads_ indicates that the GA adsorbs mainly via physical adsorption on the metal surface.
- The SEM analysis revealed that in the presence of GA the protective layer on the metal surface becomes more compact and dense with an increase in CO_2_ partial pressure. Also, the SEM analysis revealed that after 168 h of immersion, in the presence of GA, the metal surface appeared to be less damaged and smother.
- The XPS results confirmed the formation of a protective layer containing GA molecules and iron oxides on the metal surface.

## Figures and Tables

**Table 1:** Electrochemical impedance parameters with and without the presence of 1.0 g L^−1^ concentrations of GA after 24 h of immersion.

*C* (g L^−1^)	*R*_s_ (Ω cm^2^)	*CPE*_f_		*R*_f_ (Ω cm^2^)	*CPE*_dl_		*R*_ct_ (Ω cm^2^)	*R*_p_ = *R*_f_ + *R*_ct_ (Ω cm^2^)	*χ*^2^ (× 10^−3^)	*IE* (%)
		Y_f_ (mΩ^−1^ s^n^ cm^−2^)	n_f_		Y_dl_ (mΩ^−1^ s^n^ cm^−2^)	n_dl_				
1 bar Blank	7.13	1.24	0.66	11.59	1.68	0.79	217.90	229.49	1.12	-
1 bar Ga	7.39	0.34	0.61	30.29	0.68	0.81	730.60	760.89	1.50	69.83
20 bar Blank	6.92	1.28	0.68	12.18	1.85	0.76	95.07	107.25	1.34	-
20 bar GA	7.32	0.10	0.78	38.89	0.55	0.82	469.90	507.79	1.28	78.68
40 bar Blank	7.49	0.15	0.70	9.44	3.99	0.78	43.87	53.31	1.11	-
40 bar GA	7.56	0.01	0.71	50.11	0.28	0.81	374.50	424.61	1.99	87.44

**Table 2:** Potentiodynamic polarization parameters obtained after 24 h of immersion without and with 1.0 g L^−1^ of GA.

C (g L^−1^)	*E*_corr_ (V)	*i*_corr_ (μA cm^−2^)	*β*_c_ (V dec^−1^)	*IE* (%)
1 bar Blank	−0.673	17.98	0.286	-
1 bar GA	−0.676	5.54	0.334	69.23
20 bar Blank	−0.696	99.90	0.311	-
20 bar GA	−0.736	24.09	0.391	75.88
40 bar Blank	−0.600	647.05	0.316	-
40 bar GA	−0.634	84.90	0.312	86.76

**Table 3:** Parameters of the Temkin’s adsorption isotherm calculated from weight loss measurements after 24 h of immersion time.

Pressure (bar)	R^2^	Slope	Intercept	*a*	*K*_ads_	Δ*G*_ads_ (kJ mol^−1^)
1	0.985	0.483	0.708	−2.38	29.10	−8.37
20	0.995	0.478	0.786	−2.41	44.09	−9.39
40	0.996	0.453	0.844	−2.50	72.97	−10.64

**Table 4:** Weight percentage of the elements calculated from EDS analyses.

Element	Weight%			
	C	O	Fe	Total
Polished	0.70	-	99.30	100
Blank (1 bar)	1.18	-	98.82	100
1.0 g L^−1^ (1 bar)	4.06	3.51	92.43	100
Blank (20 bar)	7.28	0.83	91.89	100
1.0 g L^−1^ (20 bar)	8.00	21.98	70.02	100
Blank (40 bar)	4.99	2.05	92.96	100
1.0 g L^−1^ (40 bar)	9.90	16.21	73.89	100

## Supplementary Materials

The following are available online at https://www.mdpi.com/1996-1944/13/19/4245/s1, Table S1: Corrosion rate and inhibition efficiency obtained from weight loss measurements for the N80 carbon steel at various concentrations of GA and CO_2_ partial pressures after 24 h of immersion time, Table S2: Comparison of reported inhibition efficiency of some other corrosion inhibitors used in a CO_2_ saturated saline solution (3.5 wt.% NaCl), Table S3: Corrosion rate and inhibition efficiency obtained from weight loss measurements for the carbon steel (N80) carried out at 1.0 g L^−1^ of GA and CO_2_ partial pressures after 168 h of immersion time at 25 °C, Table S4: XPS analysis of sample steel surface after 24 h of immersion in test solution at $P_{{\mathrm{CO}}_{2}}$ = 40 bar and at 25 °C in the presence of 1.0 g L−1 of GA.
