# Supplementary material for: Effect of CO2 Partial Pressure on the Corrosion Inhibition of N80 Carbon Steel by Gum Arabic in a CO2-Water Saline Environment for Shale Oil and Gas Industry
Source: Materials (Basel). 2020 Sep 23;13(19):4245. doi: 10.3390/ma13194245 (PMC7579346; doi:10.3390/ma13194245)
Supplement: Supplementary file 1 [file materials-13-04245-s001.pdf]

Supplementary materials

Effect of CO_2 partial pressure on the corrosion inhibition of N80 carbon steel by gum arabic in a CO_2-water saline environment for shale oil and gas industry

Gaetano Palumbo[1*], Kamila Kollbek[2], Roma Wirecka[2,3], Andrzej Bernasik[3], Marcin Górny[4]

[1] Department of Chemistry and Corrosion of Metals, Faculty of Foundry Engineering, AGH University of Science and Technology, Krakow 30-059, Poland

[2] Academic Centre for Materials and Nanotechnology, AGH University of Science and Technology, Mickiewicza St. 30, 30-059 Kraków, Poland

[3] Department of Condensed Matter Physics, Faculty of Physics and Applied Computer Science, AGH University of Science and Technology, Mickiewicza St. 30, 30-059 Krakow, Poland

[4] Department of Cast Alloys and Composites Engineering, Faculty of Foundry Engineering, AGH University of Science and Technology, Krakow 30-059, Poland

* Correspondence: gpalumbo@agh.edu.pl; Tel.: +48 12 888 27 63

Table S1 Corrosion rate and inhibition efficiency obtained from weight loss measurements for the N80 carbon steel at various concentrations of GA and CO_2 partial pressures after 24 h of immersion time.

C_{inh} ($g\ L^{-1}$)	Corrosion Rate ($mm\ y^{-1}$)			*IE* (%)		
	1 bar	20 bar	40 bar	1 bar	20 bar	40 bar
			25 °C			
Blank	1.28 ± 0.18	5.86 ± 0.68	10.95 ± 0.85	-	-	-
0.6	0.51 ± 0.08	1.87 ± 0.25	2.80 ± 0.46	60.30	68.12	74.45
0.8	0.44 ± 0.06	1.54 ± 0.37	2.22 ± 0.43	65.57	73.66	79.74
1.0	0.37 ± 0.09	1.24 ± 0.33	1.69 ± 0.46	71.09	78.77	84.53
2.0	0.38 ± 0.08	1.25 ± 0.39	1.70 ± 0.25	70.31	78.66	84.47
			60 °C			
Blank	4.57±0.45	18.25±1.05	33.17±1.28	-	-	-
0.6	2.59±0.17	8,10±0.55	12.31±0.71	43.33	55.60	62.88
0.8	2.21±0.18	7.04±048	10.25±0.51	51.56	61.42	69.10
1.0	1.84±0.19	5.82±0.59	9.46±0.78	59.84	68.13	71.47
2.0	1.86±0.25	5.86±0.41	8.11±0.65	59.33	67.88	75.56

Table S2 Comparison of reported inhibition efficiency of some other corrosion inhibitors used in a CO_2 saturated saline solution (3.5 wt.% NaCl).

Inhibitors	Metal Substrate	pCO_2 (bar)	C_{inh} ($g\ L^{-1}$)	*T* (ºC)	Time (h)	*IE* (%)	Reference
Benzimidazole derivatives	J55	60	0.4	60	24	83.6	[1]
Berberine extract	C11O<br>P11OSS	60	1.0	120	168	60<br>78	[2]

	N80					92	
Momordica charantia	P11OSS	60	1.0	25	-	89	[3]
Thiourea, Mercaptoethanol	Q235	1	-	60	72	77.5<br>77.6	[4]
Gingko Biloba	J55	1	1.0	25	-	97	[5]
Chitosan, Carboxymethyl cellulose, Commercial inhibitor	API 5L X60	1	0.1	25/60	24	45/35<br>39/48<br>88/87	[6]
Chitosan Schiff Base	J55	60	0.15	65	1	95.2	[7]
Synthesized Guar gum and methylmethacrylate	P110	60	0.4	50	-	90	[8]
Imidazoline-Based	X52	60	0.1	60	24	93	[9]
Gum arabic	N80	40	1.0	25<br>60	24	84.53<br>71.47	Present study

Table S3 Corrosion rate and inhibition efficiency obtained from weight loss measurements for the carbon steel (N80) carried out at 1.0 g L^{-1} of GA and CO_2 partial pressures after 168 h of immersion time at 25 °C.

C_{inh} (g L^{-1})	Corrosion Rate (mm y^{-1})			*IE* (%)		
	**1 bar**	**20 bar**	**40 bar**	**1 bar**	**20 bar**	**40 bar**
Blank	1.88 ± 0.84	6.53 ± 0.81	11.59 ± 0.88	-	-	-
1.0	0.73 ± 0.13	1.86 ± 0.27	2.85 ± 0.55	61.41	71.53	75.41

Table S 4 XPS analysis of sample steel surface after 24 h of immersion in test solution at P_{CO_2}= 40 bar and at 25 °C in the presence of 1.0 g L^{-1} of GA .

**Peak Assignment**	**Gum arabic**		**Adsorbed gum arabic**	
	**Binding Energy (eV)**	**Atomic concentration (%)**	**Binding Energy (eV)**	**Atomic concentration (%)**
O1s	-	33.6	-	42.9
O^{2-}	-	-	529.7	28.7
O-C	531.3	16.0	531.2	32.2
O-C-O/O=C	532.6	84.0	532.7	39.1
C1s	-	61.6	-	48.8
C-C/C-H	284.8	25.3	284.8	40.3
C=O/C-OH/C-N	286.3	54.7	286.3	38.5
O-C-O/N-C=O	288.0	20.0	287.9	21.2

Fe2p	-	-	-	4.9
Fe^{3+} (Fe_2O_3)	-	-	710.5	47.1
	-	-	713.0	15.2
Sat	-	-	719.0	14.9
$Fe2p_{1/2}$	-	-	-	22.8
N1s	-	4.8	-	2.4
N-C	400.00	100	399.8	71.5
N-Fe	-	-	397.6	28.5

References

1. Singh, A.; Ansari, K.R.; Quraishi, M.A.; Lgaz, H., Effect of Electron Donating Functional Groups on Corrosion Inhibition of J55 Steel in a Sweet Corrosive Environment: Experimental, Density Functional Theory, and Molecular Dynamic Simulation. *Materials* **2019,** 12, (1), 17.
2. Lin, Y.; Singh, A.; Ebenso, E.E.; Quraishi, M.A.; Zhou, Y.; Huang, Y., Use of HPHT Autoclave to Determine Corrosion Inhibition by Berberine extract on Carbon Steels in 3.5% NaCl Solution Saturated with CO2 *Int. J. Electrochem. Sci.* **2015**, *10*, 194–208.
3. Singh, A.; Lin, Y.; Liu, W.; Ebenso, E.E.; Pan, J., Extract of Momordica charantia (Karela) Seeds as Corrosion Inhibitor for P110SS Steel in CO2 Saturated 3.5% NaCl Solution. *Int. J. Electrochem. Sci.* **2013**, *8*, 12884–12893.
4. Tang, J.; Hu, Y.; Han, Z.; Wang, H.; Zhu, Y.; Wang, Y.; Nie, Z.; Wang, Y., Experimental and Theoretical Study on the Synergistic Inhibition Effect of Pyridine Derivatives and Sulfur-Containing Compounds on the Corrosion of Carbon Steel in CO2-Saturated 3.5 wt.% NaCl Solution *Molecules* **2018,** 23, (12), 3270.
5. Singh, A.; Lin, Y.; Ebenso, E.E.; Liu, W.; Pan, J.; Huang, B., Gingko biloba fruit extract as an eco-friendly corrosion inhibitor for J55 steel in CO2 saturated 3.5% NaCl solution. *J. Ind. Eng. Chem.* **2015**, *24*, 219–228.
6. Umoren, S.A.; AlAhmary, A.A.; Gasem, Z.M.; Solomon, M.M., Evaluation of chitosan and carboxymethyl cellulose as ecofriendly corrosion inhibitors for steel. *Int. J. Biol. Macromol.* **2018**, *117*, 1017–1028.
7. Ansari, K.R.; Chauhan, D.S.; Quraishi, M.A.; Mazumder, M.A.J.; Singh, A., Chitosan Schiff base: an environmentally benign biological macromolecule as a new corrosion inhibitor for oil & gas industries. *Int. J. Biol. Macromol.* **2020**, *144*, 305–315.
8. Singh, A.; Ansari, K.R.; Quraishi, M.A., Inhibition effect of natural polysaccharide composite on hydrogen evolution and P110 steel corrosion in 3.5 wt% NaCl solution saturated with CO2: Combination of experimental and surface analysis. *International Journal of Hydrogen Energy* **2020**.
9. Mustafa, A.H.; Ari-Wahjoedi, B.; Ismail, M.C., Inhibition of CO2 Corrosion of X52 Steel by Imidazoline-Based Inhibitor in High Pressure CO2-Water Environment. *J. Mater. Eng. Perform.* **2013,** 22, (6), 1748–1755.
